# Randomized trial of tofacitinib in active ulcerative colitis: analysis of efficacy based on patient-reported outcomes

**DOI:** 10.1186/s12876-015-0239-9

**Published:** 2015-02-05

**Authors:** Julián Panés, Chinyu Su, Andrew G Bushmakin, Joseph C Cappelleri, Carla Mamolo, Paul Healey

**Affiliations:** 1 Hospital Clínic de Barcelona. IDIBAPS, CIBERehd, Barcelona, 08036 Spain; 2 Pfizer Inc, Collegeville, PA USA; 3 Pfizer Inc, Groton, CT USA

**Keywords:** Ulcerative colitis, Patient reported outcomes, Quality of life, Tofacitinib

## Abstract

**Background:**

Tofacitinib, a novel, oral Janus kinase inhibitor, demonstrated a dose-dependent efficacy for induction of clinical response and remission in patients with active ulcerative colitis (UC). The objective of the current study was to determine the effect of tofacitinib on patient-reported outcomes (PROs).

**Methods:**

Eligible patients (≥18 years of age) with a diagnosis of active UC (total Mayo score of 6-12 points and moderately-to-severely active disease on sigmoidoscopy) were randomized in a 2:2:2:3:3 ratio to receive oral tofacitinib 0.5 mg, 3 mg, 10 mg, or 15 mg, or placebo twice daily (BID) for 8 weeks. PROs were assessed by the Inflammatory Bowel Disease Questionnaire (IBDQ) and the Inflammatory Bowel Disease Patient-Reported Treatment Impact (IBD PRTI) survey.

**Results:**

At Week 8, mean IBDQ total scores had improved relative to baseline across all five treatment groups (baseline range 123.2-134.5; Week 8 range 149.6-175.4). Improvement from baseline was significantly greater (*P* = 0.001) for tofacitinib 15 mg BID versus placebo. For tofacitinib 15 mg BID, most patients reported satisfaction or extreme satisfaction, definite preference for tofacitinib, and definite willingness to use tofacitinib again on the IBD PRTI at week 8. Patients achieving endoscopic remission (Mayo endoscopy score of 0) had significantly higher IBDQ scores and favorable PRTI scores than those not achieving endoscopic remission.

**Conclusions:**

Short-term treatment with tofacitinib BID was associated with dose-dependent improvement in health-related quality of life and patient preferences for tofacitinib. The results complement previously reported efficacy and safety data for the Phase II study. (NCT 00787202, November 6, 2008).

**Electronic supplementary material:**

The online version of this article (doi:10.1186/s12876-015-0239-9) contains supplementary material, which is available to authorized users.

## Background

In North America the highest annual incidence of UC is reported to be 19.2 per 100,000 person-years, in Europe 24.3, and 6.3 in Asia and the Middle East [1]. Current medical therapies for ulcerative colitis (UC) are only effective in a proportion of patients and for patients who fail to respond, surgery is often necessary. A study of Canadian patients with UC calculated approximately one-tenth of patients will have a colectomy within 10 years of diagnosis and one-sixth within 20 years of diagnosis [2].

Tofacitinib (CP-690,550) is a novel, oral small molecule Janus kinase (JAK) inhibitor that is being investigated for use in several inflammatory diseases, including UC. In kinase assays, tofacitinib preferentially inhibits signaling by receptors associated with JAK3 and JAK1 while it demonstrates reduced inhibition for JAK2- and tyrosine kinase 2 (TYK2)-associated signaling [3]. JAKs mediate signal transduction activity by the surface receptors for multiple cytokines. Importantly, tofacitinib directly inhibits signaling for an important subset of pro-inflammatory cytokines including IL-2, -4, -7, -9, -15, and -21 [3,4]. These cytokines are integral to lymphocyte activation, proliferation, and function, and inhibition of their signaling may result in modulation of multiple aspects of the immune response [3-6].

In an 8-week Phase II randomized controlled trial (RCT) in patients with moderately-to-severely active UC (clinicaltrials.gov NCT00787202), treatment with tofacitinib was associated with a significant dose-dependent improvement in clinical response and clinical remission compared with placebo. Definitions of remission and response have been described previously [7].

Patient-reported outcomes (PROs) represent an integral and increasingly important aspect of drug development as a complementary measure of efficacy because they represent direct measures of patient-perceived benefit. The Inflammatory Bowel Disease Questionnaire (IBDQ) has been used in many trials and is generally accepted as providing valuable additional information regarding the efficacy of treatment for patients with UC. The outcome can be measured in absolute terms (e.g., severity of a symptom, sign, or state of the disease) or as a change from a previous measure. The Inflammatory Bowel Disease Patient-Reported Treatment Impact (IBD PRTI) survey asks about patient’s overall satisfaction with their current treatment and overall willingness to initiate and continue the treatment versus their previous treatment.

To evaluate the effect of tofacitinib on health-related quality of life (HRQoL) and patient preferences for tofacitinib, we report for the first time a distinct set of PROs from the 8-week Phase II RCT. We further report on the relationship between the PROs of the IBDQ and the IBD PRTI. The relationship between these PROs and the Mayo score-based clinical and endoscopic outcomes were also analyzed.

## Methods

### Patients

Eligible patients were ≥18 years of age and had a diagnosis of UC for ≥3 months before study entry, had active disease defined by a Mayo score of 6-12 points (in which scores range from 0 to 12 with higher scores indicating more severe disease) [8], and moderately-to-severely active disease on sigmoidoscopy. Patients could receive oral mesalamine and/or oral corticosteroids at a stable dose of ≤30 mg per day of prednisone equivalence. Exclusion criteria, permitted medications, and prohibited concomitant medications have previously been described [7].

### Study design

This 8-week, multicenter, Phase II RCT was conducted at 51 centers in 17 countries between January 2009 and September 2010. Patients were randomly assigned in a 2:2:2:3:3 ratio to receive oral tofacitinib at doses of 0.5 mg, 3 mg, 10 mg, or 15 mg, or placebo administered twice daily (BID). Patients were treated for 8 weeks and followed for a further 4 weeks. The randomization process and primary efficacy and safety evaluations have previously been described in full [7].

### PRO instruments

PROs were assessed by the IBDQ and the 3-item IBD PRTI (Version 2) survey. These PROs were pre-defined secondary efficacy measures in the study protocol. The IBDQ is a psychometrically validated instrument for measuring disease-specific HRQoL in patients with IBD, including UC [9]. The IBDQ comprises 32 items which are grouped into four domains: bowel function, emotional status, systemic symptoms, and social function. The four domains are scored as follows: bowel symptoms, 10 to 70; systemic symptoms, 5 to 35; emotional function, 12 to 84; and social function: 5 to 35. With 1 to 7 points for each item, the total score on this index ranges from 32 to 224, with higher scores indicating better HRQoL [10]. IBDQ response has been defined as an increase in IBDQ total score of ≥16 points from baseline, and remission has been defined as a total IBDQ score of ≥170 points [9]. The IBDQ was self-completed by patients at baseline (Day 1) and at Week 8.

The IBD PRTI survey (Additional file 1) comprises three individual questions concerning patient satisfaction with study treatment, patient preference for study drug over prior treatment (this question on subject preference for study medication is prefaced by a simple question about previous treatments for IBD in order to place the preference question into context), and patient willingness to reuse the study treatment again. Each of the three questions (the fourth question on previous treatment was for information only) was scored on a 5-point scale. Patients completed the survey at the end of the study (Week 8), or at early withdrawal from the study. The IBD PRTI is a measure for assessing patient satisfaction, patient preferences for study treatments, and willingness to use treatment again. The utility of assessing these concepts within clinical trials has been demonstrated by several authors [11-14].

### Clinical evaluation

As previously reported [7], disease activity of patients with UC was measured using the Mayo scoring system [8]. The full Mayo system consists of four subscores, each graded from 0 to 3 points, with higher scores indicating more severe disease. Total scores per patient range from 0 to 12 points. The four subscores are: stool frequency, rectal bleeding, findings of flexible sigmoidoscopy, and physician global assessment. Clinical response was defined as a decrease in Mayo score from baseline of ≥3 points and ≥30%, with an accompanying decrease in the subscore for rectal bleeding of ≥1 point or an absolute subscore for rectal bleeding of 0 or 1. Clinical remission was defined as a total Mayo score of ≤2 points, with no individual subscore exceeding 1 point, and endoscopic remission was defined as a Mayo endoscopic subscore of 0. The partial Mayo score is a non-invasive outcome measure based on three of the four full Mayo subscores and lacks the endoscopy findings subscore; therefore, it is based on a scale of 0 to 9.

During the trial, the total Mayo score was assessed at baseline and Week 8 and the partial Mayo score was assessed at baseline and Weeks 2, 4, and 8.

### Statistical analysis

#### Pre-specified

The primary efficacy endpoint of the Phase II study was based on the total Mayo score clinical response at Week 8. The IBDQ, IBD PRTI, and partial Mayo score we report were pre-specified secondary efficacy analyses.

The analysis of clinical response and clinical remission at Week 8 was performed using a maximal efficacy (E_max_) model with treatment group as a factor and a term included for prior anti-tumor necrosis factor therapy. Patients with data missing for reasons other than insufficient response to therapy or an adverse event related to UC were excluded from the analysis. All IBDQ data for the four domains and total score were summarized by time post-dose for each dose. The Week 8 change from baseline for each dose was analyzed using an analysis of covariance that allowed for variation due to dose group and baseline value. The individual IBD PRTI items were scored separately. Missing IBDQ and IBD PRTI data were not replaced.

The full analysis set (FAS) was the primary analysis set for all efficacy analyses and included all randomized patients who had either withdrawn as a treatment failure or had completed at least 1 week of dosing and had at least one valid Mayo score during the active double-blind phase of the study. The intention-to-treat population analysis classified all patients with missing Week 8 data as non-responders.

#### Post-Hoc analyses

We performed a series of supplemental (post-hoc) calculations using all available data (by pooling all treatment groups) to evaluate relationships between the IBDQ, the Mayo scale (full and partial),the Mayo endoscopy subscore, and the IBD PRTI survey. No imputation of missing data was performed.

Relationships between the IBDQ and the Mayo scores were examined using a repeated measures longitudinal model [15,16]. The Mayo score was used as a continuous predictor in the modeling. By doing so, the linear relationship was effectively imposed between the outcome and predictor. In another series of models, considered as sensitivity analyses, the Mayo score was used as a categorical predictor. This approach did not impose any functional relationship between the predictor and outcome. If a linear or approximately linear relationship was realized between the IBDQ and the Mayo score, the Mayo score as a continuous predictor would be more sensitive and more easily interpretable. The relationship between the IBD PRTI score and the Mayo score at Week 8 was examined using a regression model. In one set of models, the Mayo score was a continuous predictor and in another set, the Mayo score was a categorical predictor.

Pearson correlations between IBDQ score and Mayo score, and between IBD PRTI and Mayo score, were calculated. A two-by-two contingency table on remission status is provided (Additional file 2).

### Ethical considerations

The study was performed in compliance with the Declaration of Helsinki and the International Conference on Harmonisation Good Clinical Practice Guidelines, and was approved by the Institutional Review Boards at each study center. All patients provided written informed consent. The full names of every Institutional review board that approved the study in each center is provided in Additional file 3.

## Results

### Demographics

Patient demographic and baseline disease characteristics have been described previously [7]. Briefly, a total of 195 patients were randomized and 194 received study drug: 48 patients in the placebo group and 31, 33, 33, and 49 in the tofacitinib groups received 0.5 mg, 3 mg, 10 mg, and 15 mg BID, respectively. A total of 157 of the 194 patients (80.9%) completed the study (Additional file 4). The one patient who did not receive the study drug (in the placebo group) was excluded from the analyses. The demographics and baseline disease characteristics of the five groups were similar, with only the proportion of patients using a glucocorticoid at baseline being significantly different (*P* < 0.05) across treatment groups [tofacitinib 0.5, 3, 10, and 15 mg BID; n = 11 (35%), 10 (30%), 19 (58%), 13 (27%), respectively, versus placebo, 13 (27%)].

### IBDQ

**Figure 1:**
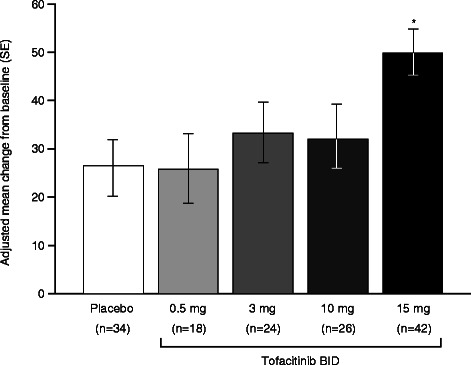
**Adjusted mean change from baseline (SE) in total IBDQ score at Week 8.** A higher score indicates better quality of life. The ANCOVA model used treatment group as a factor and the baseline value as a covariate. The center was not included in the model. *p = 0.001 compared with placebo (analysis of covariance). BID, twice daily; IBDQ, inflammatory bowel disease questionnaire; SE, standard error.

Mean IBDQ total scores (FAS, non-imputation population) at baseline were similar between treatment groups, ranging from 123.8 to 134.5 in the tofacitinib groups (123.2 in the placebo group). At Week 8, mean IBDQ total scores had improved (higher scores indicate better quality of life) across all treatment groups compared to baseline, ranging from 149.6-175.4 in the tofacitinib groups (151.3 in the placebo group) (Table 1). The adjusted mean change (improvement) from baseline was significantly greater (*P* = 0.001) at Week 8 for tofacitinib 15 mg BID versus placebo (Figure 1). A trend towards a dose-response was observed in the IBDQ scores.

**Table 1:** **IBDQ total scores (FAS), IBDQ domain score (FAS), and percentage of patients achieving clinically meaningful IBDQ response and remission (ITT)**

	Placebo (N = 48)	Tofacitinib BID			
		0.5 mg (N = 31)	3 mg (N = 33)	10 mg (N = 33)	15 mg (N = 49)
**IBDQ total score (SD)**					
Baseline, n	47	31	30	31	48
Mean	123.2 (29.5)	123.8 (34.5)	132.3 (33.6)	134.5 (32.5)	124.0 (34.9)
Week 8, n	34	18	26	28	43
Mean	151.3 (33.4)	149.6 (37.7)	166.6 (42.4)	160.5 (33.7)	175.4 (35.3)
Mean change from baseline	27.8 (29.8)	27.7 (33.4)	30.3 (27.3)	30.4 (39.8)	50.7 (35.6)^#^
**Mean change from baseline in IBDQ domains (SD)**					
Week 8, n	34	18	24	26	42
Bowel function	9.15 (10.59)	11.06 (10.48)	11.46 (9.65)	13.19 (14.18)	18.96 (11.60)^#^
Emotional status	9.49 (11.89)	6.33 (14.09)	9.21 (10.60)	7.88 (14.70)	16.48 (14.75)^#^
Systemic symptoms	4.44 (5.86)	4.89 (5.81)	4.79 (4.62)	3.85 (6.39)	7.24 (5.88)^#^
Social function	4.67 (6.49)	5.44 (7.45)	4.83 (5.72)	5.46 (6.96)	8.04 (7.07)^#^
**IBDQ response** ^**a**^ **/remission** ^**b**^					
Week 8, n	48	31	33	33	49
Patients with Clinically meaningful IBDQ response, n (%)	20 (41.7)	12 (38.7)	14 (42.4)	16 (48.5)	37 (75.5)
Logistic regression odds ratio^a^ (95% CI)	Referent	0.84 (0.33–2.13)	1.05 (0.42–2.60)	1.27 (0.52–3.11)	4.18* (1.75–10.02)
Patients with IBDQ remission, n (%)	11 (22.9)	8 (25.8)	14 (42.4)	12 (36.4)	30 (61.2)
Logistic odds ratio^c^ (95% CI)	Referent	1.12 (0.39–3.23)	2.56 (0.96–6.80)	1.87 (0.70–4.99)	5.23** (2.14–12.75)

Range of IBDQ total score = 32-224. Maximal domain score: Bowel function = 70; Emotional status = 84; Systemic symptoms = 35; Social function = 35.

^#^*P* <0.05 versus placebo (ANCOVA).

*P = 0.001, **P < 0.001: odds ratio versus placebo (logistic regression).

^a^Clinically meaningful IBDQ response = ≥16 point improvement in total IBDQ score.

^b^IBDQ remission = total IBDQ score of ≥170 at Week 8.

^c^versus placebo.

BID, twice daily; CI, confidence interval; FAS, full analysis set; IBDQ, Inflammatory Bowel Disease Questionnaire; ITT, intention-to-treat population where patients with missing Week 8 data were treated as non-responders; NA, not applicable.

Statistically significant (*P* < 0.05) improvements in mean change from baseline were observed across all four IBDQ domains, with the greatest improvements in patients in the tofacitinib 15 mg BID group (Table 1). A logistic regression analysis showed an odds ratio of 4.18 (95% confidence interval [CI], 1.75–10.02) for patients receiving tofacitinib 15 mg BID to achieve a clinically meaningful IBDQ response (≥16-point improvement in total IBDQ score) at Week 8 versus placebo. Similarly, the odds ratio over the same time period for patients achieving IBDQ remission (total IBDQ score of ≥170) was also statistically significant for tofacitinib 15 mg BID (5.23 [95% CI, 2.14–12.75]; *P* < 0.001) (Table 1).

### IBD PRTI

**Figure 2:**
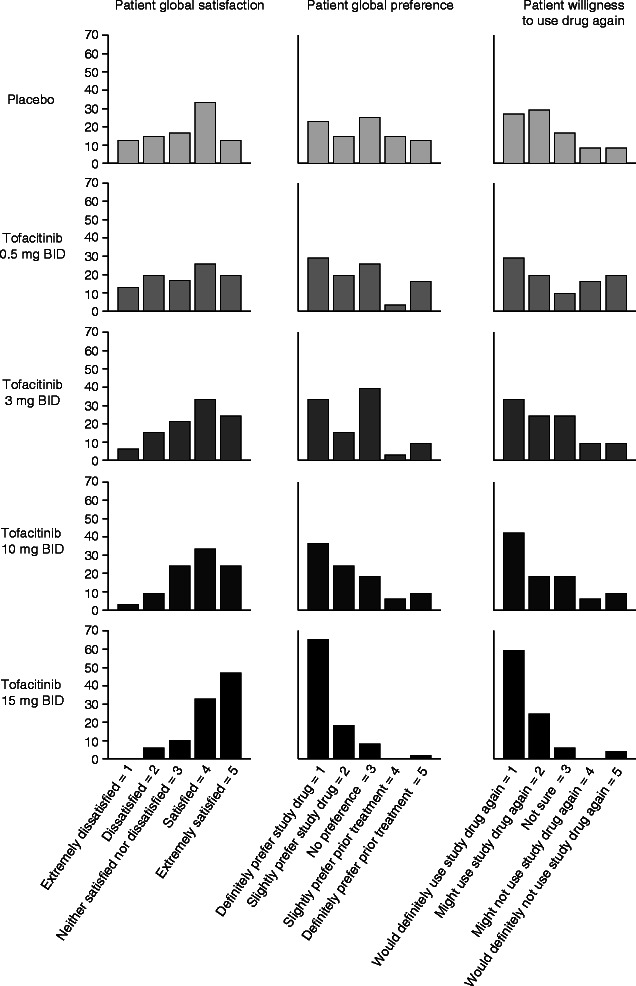
**IBD PRTI score frequency distributions at Week 8 (FAS).** BID, twice daily; PRTI, IBD patient-reported treatment impact (version 2) questionnaire.

For each of the three component items of the IBD PRTI (patient global satisfaction, patient global preference, and patient willingness to use the drug again), dose-related improvements were observed that were greatest in patients receiving tofacitinib 15 mg BID (Table 2). IBD PRTI component frequency distributions at Week 8 also highlighted treatment-related preferences for tofacitinib such that, for tofacitinib 15 mg BID, the majority of patients reported satisfaction or extreme satisfaction, a definite preference for tofacitinib over previous treatments, and a definite willingness to use tofacitinib again (Figure 2).

**Table 2:** **IBD PRTI scores at Week 8 (observed population**)

IBD PRTI component score, mean (SE)	Placebo (n = 43)	Tofacitinib BID			
		0.5 mg (n = 29)	3 mg (n = 33)	10 mg (n = 31)	15 mg (n = 47)
Patient global satisfaction^a^	3.2 (0.2)	3.2 (0.3)	3.5 (0.2)	3.7 (0.2)	4.3 (0.1)
Patient global preference^b^	2.8 (0.2)	2.6 (0.3)	2.4 (0.2)	2.2 (0.2)	1.5 (0.1)
Patient willingness to use drug again^c^	2.3 (0.2)	2.8 (0.3)	2.4 (0.2)	2.2 (0.2)	1.6 (0.1)

^a^Extremely dissatisfied with study drug = 1, Dissatisfied with study drug = 2, Neither satisfied nor dissatisfied with study drug = 3, Satisfied with study drug = 4, Extremely satisfied with study drug = 5.

^b^Definitely prefer study drug over prior treatment = 1, Slightly prefer study drug = 2, No preference = 3, Slightly prefer prior treatment = 4, Definitely prefer prior treatment = 5.

^c^Would definitely use study drug again = 1, Might use study drug again = 2, Not sure = 3, Might not use study drug again = 4, Would definitely not use study drug again = 5.

BID, twice daily; PRTI, IBD Patient-Reported Treatment Impact (Version 2) survey; SE, standard error.

### Correlation between PROs and Mayo score based outcomes

**Figure 3:**
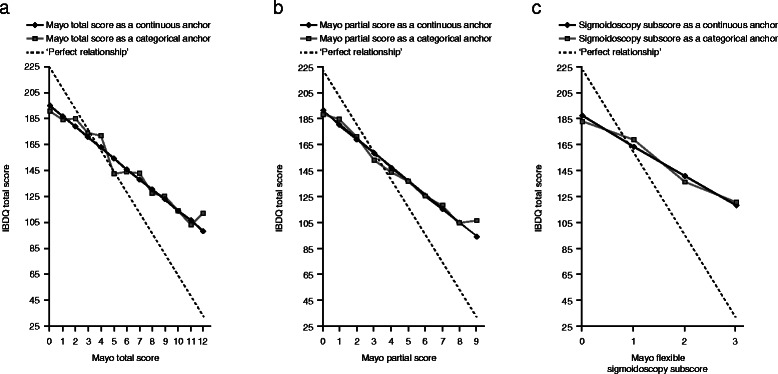
**Relationship between the IBDQ total score (all patients) versus the total Mayo score (a), the Mayo partial score (b), and the Mayo endoscopic subscore (c).**

The correlations between the IBDQ total score versus the total Mayo score, the partial Mayo score, and the Mayo endoscopic subscore were all consistent, in the expected direction (with a lower Mayo score corresponding to a higher IBDQ score), and approximately linear (Figure 3A-C). A 1-point change on the total Mayo score corresponded, on average, to a change of 8.0 (*P* < 0.0001) points on the IBDQ total score. The correlation at baseline was 0.33, and at Week 8, 0.62 (*P* < 0.0001) (Figure 3A). A 1-point change on the partial Mayo score corresponded, on average, to a change of 10.8 (*P* < 0.0001) points on the IBDQ total score. The correlation at baseline was 0.32, and at Week 8, 0.64 (*P* < 0.0001) (Figure 3B). Patients achieving endoscopic remission (Mayo endoscopy score of 0) had an IBDQ total score significantly higher (*P* < 0.0001) than patients without endoscopic remission. The differences in the IBDQ total scores between patients with, versus patients without, endoscopic remission were 45.7 when a linear relationship was imposed in modeling and 41.8 when the Mayo endoscopy score was used as a categorical predictor (Figure 3C). Correlation at baseline was small (0.17), and at Week 8, moderate (0.46, *P* < 0.0001). . We found a significant correlation between the bowel domain subscore of IBDQ and the two domains of the Mayo score considered as PROs: stool frequency (r = -0.55, p < 0.0001), rectal bleeding score (r = -0.56, p < 0.0001), and the combined stool frequency and rectal bleeding score (r = -0.64, p < 0.0001) at week 8. The corresponding correlations at week 0 were smaller (r = 0.31. r = 0.27 and r = 0.39 respectively, all p < 0.001) due to the restricted range of data.

**Figure 4:**
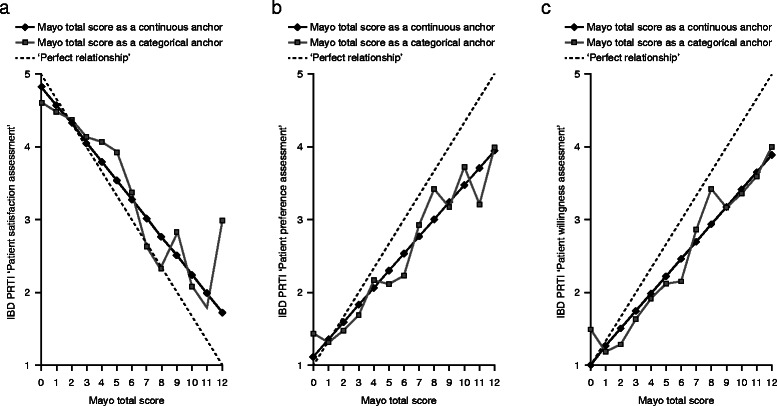
**Relationship between the components of the IBD PRTI (all patients) including patient satisfaction (a), preference (b), and willingness (c) versus the total Mayo score.** IBD PRTI item scores: Patient satisfaction assessment; Extremely dissatisfied with study drug = 1, Dissatisfied with study drug = 2, Neither satisfied nor dissatisfied with study drug = 3, Satisfied with study drug = 4, Extremely satisfied with study drug = 5. Patient preference assessment; Definitely prefer study drug over prior treatment = 1, Slightly prefer study drug = 2, No preference = 3, Slightly prefer prior treatment = 4, Definitely prefer prior treatment = 5. Patient willingness assessment; Would definitely use study drug again = 1, Might use study drug again = 2, Not sure = 3, Might not use study drug again = 4, Would definitely not use study drug again = 5.

**Figure 5:**
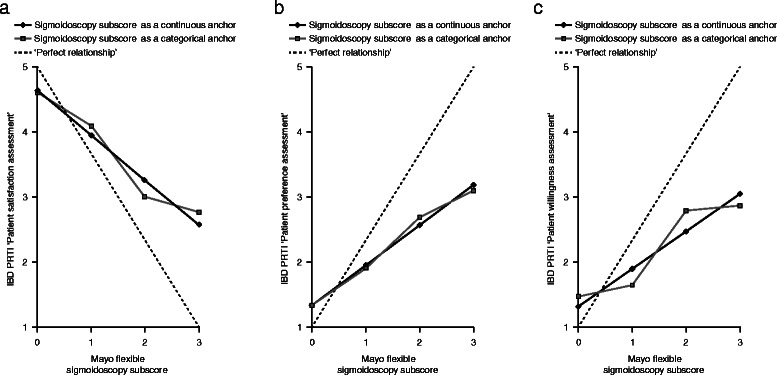
**Relationship between the components of the IBD PRTI (all patients) including patient satisfaction (a), preference (b), and willingness (c) versus the Mayo endoscopy score.** IBD PRTI item scores: Patient satisfaction assessment; Extremely dissatisfied with study drug = 1, Dissatisfied with study drug = 2, Neither satisfied nor dissatisfied with study drug = 3, Satisfied with study drug = 4, Extremely satisfied with study drug = 5. Patient preference assessment; Definitely prefer study drug over prior treatment = 1, Slightly prefer study drug = 2, No preference = 3, Slightly prefer prior treatment = 4, Definitely prefer prior treatment = 5. Patient willingness assessment; Would definitely use study drug again = 1, Might use study drug again = 2, Not sure = 3, Might not use study drug again = 4, Would definitely not use study drug again = 5.

Furthermore, when the IBD PRTI components for all patients were modeled against the total Mayo score (Figure 4A-C), the partial Mayo score (Additional file 5), and the Mayo endoscopy subscore (Figure 5A-C) all correlations were consistent, in the right direction, and approximately linear. For the total Mayo score, a 1-point change corresponded to a mean change in Patient Preference Assessment of 0.24 points (*P* < 0.0001), 0.26 points in Patient Satisfaction Assessment (*P* < 0.0001) and 0.24 points in Patient Willingness Assessment (*P* < 0.0001) if a model with imposed linear relationship between IBD PRTI item score and the total Mayo score is used. Correlation at Week 8 between the IBD PRTI item scores and the total Mayo score were moderate-to-high: 0.59 (*P* < 0.0001; Patient Preference Assessment), 0.70 (p < 0.0001; Patient Satisfaction Assessment), and 0.60 (p < 0.0001; Patient Willingness Assessment) (Figure 4A-C).

For partial Mayo score, a 1-point change corresponded to a mean change of: 0.31 points (P < 0.0001; patient preference assessment), 0.34 points (P < 0.0001; patient satisfaction assessment), and 0.32 points (P < 0.0001; patient willingness assessment) if a model with imposed linear relationship between the IBD PRTI item score and the partial Mayo score was used. Correlation at Week 8 between IBD PRTI item scores and the partial Mayo were moderate to high: 0.58 (P < 0.0001; patient preference assessment), 0.69 (P < 0.0001; patient satisfaction assessment), and 0.60 (P < 0.0001; patient willingness assessment) (Additional file 5).

Patients achieving endoscopic remission (Mayo endoscopy score of 0) were significantly different from other patients (Mayo endoscopy score of 1, 2, or 3) on all IBD PRTI components. Those differences were: 1.2 points (*P* < 0.0001; Patient Preference Assessment), 1.4 points (*P* <0.0001; Patient Satisfaction Assessment), and 1.2 points (*P* < 0.0001; Patient Willingness Assessment) if a model with imposed linear relationship between IBD PRTI item score and Mayo endoscopy score was used. If a model with Mayo endoscopy score as a categorical predictor was used, those differences were: 1.2 points (*P* < 0.0001; Patient Preference Assessment), 1.3 points (*P* < 0.0001; Patient Satisfaction Assessment), and 1.0 points (*P* < 0.0001; Patient Willingness Assessment). Correlations at Week 8 between IBD PRTI item score and Mayo endoscopy score were moderate: 0.47 (*P* < 0.0001; Patient Preference Assessment), 0.56 (*P* < 0.0001; Patient Satisfaction Assessment), and 0.44 (*P* < 0.0001; Patient Willingness Assessment) (Figure 5).

## Discussion

We have reported the first detailed and comprehensive set of PRO results on the use of tofacitinib in patients with moderately-to-severely active UC. The results showed that short-term treatment with tofacitinib was associated with statistically significant improvement in HRQoL and patient preferences for the study medication compared with placebo. Improvements in HRQoL were dose-dependent and, at the maximum dose of tofacitinib examined (15 mg BID), the adjusted mean change in IBDQ total score between baseline and Week 8 was statistically significantly greater compared with placebo. These results support the clinical efficacy data reported previously from this Phase II study, whereby patients with moderately-to-severely active UC treated with tofacitinib were more likely to achieve clinical response and remission, and endoscopic response and remission, than those receiving placebo [7]. Our results are consistent with significant improvements in PROs for patients treated with tofacitinib reported from RCTs in other chronic inflammatory conditions, such as rheumatoid arthritis [17,18] and psoriasis [19].

### Relationship between PROs and the Mayo scores

The observed approximately linear relationship between the PROs of the IBDQ and the IBD PRTI with the Mayo scoring system for assessing clinical response demonstrated the clinical relevance of the IBDQ and the IBD PRTI in patients with moderately-to-severely active UC. Interestingly, we also observed statistically significant moderate to large correlations between the two domains of Mayo score being considered as PROs, namely stool frequency and rectal bleeding, and the IBDQ bowel domain subscore. Relationships between Mayo scores and both PRO measurements were stable, in the right direction (reflecting benefit to the patient), and approximately linear. Disease severity is reportedly the most important factor influencing HRQoL in patients with UC [20,21], and the results we report provide quantitative evidence that reducing the signs and symptoms of UC with tofacitinib treatment improves patients’ HRQoL. These results also prove the responsiveness of IBDQ score and PRTI items to therapeutic interventions. The relationships also provide an estimate of the magnitude of improvement; for example, a 1-point change on the total Mayo score corresponded, on average, to a change (improvement) of 8.0 (*P* < 0.0001) points in the total IBDQ score. Furthermore, our results also suggest mucosal healing should be a therapeutic objective in patients with UC as patients achieving endoscopic remission (Mayo endoscopy subscore of 0) had significantly different IBDQ and IBD PRTI component scores compared with patients with persistent endoscopic disease activity.

Our results further support the use of PROs for assessment of drug efficacy. However, the low specificity of the IBDQ at the individual level to identify patients achieving remission defined by the Mayo score, indicate that PROs could be used as complementary endpoints to endoscopic or composite (clinical and endoscopic) scores.

### IBD PRTI

Patient choice of treatment is a central feature of IBD healthcare, and the IBD PRTI survey was specifically designed to determine a patient’s treatment preference. We are not aware of any existing IBD instrument/tool that captures the important outcome of patient preference, and as such, the IBD PRTI may have a future role as a useful indicator of patient preference. We believe the trial we report is the first trial in patients with UC to incorporate this particular PRO and it was interesting to observe the approximate linear associations of the IBDQ with the Mayo scoring system were also replicated by the associations of the IBD PRTI survey with the Mayo scoring system. The development of novel PRO tools for investigating UC is warranted because current outcomes capture only a portion of the impact of the disease on patients’ lives and may not accurately measure a patient’s experience with UC [22].

### Study limitations

The limitations of our analyses are that they were based on a relatively small sample size from a single clinical trial that focused on patients with moderately-to-severely active disease. Also, the duration of the study was short, and the long-term benefit of tofacitinib on PROs remains to be evaluated. Future analyses from larger ongoing Phase III RCTs in UC (ClinicalTrials.gov identifiers: NCT01470612, NCT01458574, NCT01465763, and NCT01458951) will be performed to corroborate and expand our findings.

## Conclusions

Short-term treatment with tofacitinib was associated with statistically significant dose-dependent improvement in HRQoL and patient preference for the study medication. The results support the clinical efficacy and safety data previously reported for this Phase II study [7] and reinforce the rationale for continued investigation into the use of tofacitinib in the treatment of patients with moderately-to-severely active UC. In utilizing clinical efficacy data, we showed a strong, approximately linear relationship between the IBDQ, the IBD PRTI, and the Mayo scoring system, and also between the IBDQ bowel function domain subscore and the IBD PRTI, thus supporting the clinical relevance and validity of these PROs. Additionally, IBDQ and IBD PRTI scores were shown to be significantly different for patients achieving endoscopic remission compared with those who did not.

## Additional files

Additional file 1: **IBD remission status (endoscopic remission vs IBDQ remission [total score ≥170]).** Relationship between endoscopic remission and IBDQ remission. Additional file 2: **Institutional review board that approved the study.** Full names of every Institutional review board that approved the study in each center. Additional file 3: **Inflammatory Bowel Disease Patient-Reported Treatment Impact.** Components of the IBD Patient-Reported Treatment Impact questionnaire. Each of the three questions (except the question on previous treatment, which was informational only) was scored on a 5-point scale. Additional file 4:
**Patient disposition. patient disposition in the study.**
Additional file 5: **Relationship between Patient-Reported Treatment Impact and clinical disease activity in ulcerative colitis.** Relationship between the components of the IBD Patient-Reported Treatment Impact (all patients) versus the Mayo partial score. IBD PRTI item scores: Patient satisfaction assessment; Extremely dissatisfied with study drug = 1, Dissatisfied with study drug = 2, Neither satisfied nor dissatisfied with study drug = 3, Satisfied with study drug = 4, Extremely satisfied with study drug = 5. Patient preference assessment; Definitely prefer study drug over prior treatment = 1, Slightly prefer study drug = 2, No preference = 3, Slightly prefer prior treatment = 4, Definitely prefer prior treatment = 5. Patient willingness assessment; Would definitely use study drug again = 1, Might use study drug again = 2, Not sure = 3, Might not use study drug again = 4, Would definitely not use study drug again = 5.

## Abbreviations

BID: Twice daily
FAS: Full analysis set
HRQoL: Health-related quality of life
IBD: Inflammatory bowel disease
IBDQ: Inflammatory bowel disease questionnaire
JAK: Janus kinase
PROs: Patient-reported outcomes
PRTI: Patient-reported treatment impact
RCT: Randomized controlled trial
UC: Ulcerative colitis
