# Supplementary material for: Randomized trial of tofacitinib in active ulcerative colitis: analysis of efficacy based on patient-reported outcomes
Source: BMC Gastroenterol. 2015 Feb 5;15:14. doi: 10.1186/s12876-015-0239-9 (PMC4323227; doi:10.1186/s12876-015-0239-9)
Supplement: Additional file 1: — IBD remission status (endoscopic remission vs IBDQ remission [total score ≥170]). Relationship between endoscopic remission and IBDQ remission. [file 12876_2015_239_MOESM1_ESM.docx]

## Additional file 1: Inflammatory Bowel Disease Patient-Reported Treatment Impact (Version 2) (IBD PRTI) Survey

The IBD PRTI questionnaire comprises 3 individual questions administered to the patient.

Each of these questions (except the question on previous treatment, which was informational only) was scored on a 5-point scale.

**(1) Patient-Reported Treatment Impact Assessment**

Overall, how satisfied are you with the drug that you received since you entered this trial?

**Please check (X) ONE only:**

□ (5) Extremely satisfied
 □ (4) Satisfied
 □ (3) Neither satisfied nor dissatisfied
 □ (2) Dissatisfied
 □ (1) Extremely dissatisfied

**(2) Patient Global Preference Assessment** (This question on patient preference for study medication is prefaced by a simple question of previous treatments for IBD received in order to place the preference question into context)

Preface question

Before enrolling in this clinical trial, what treatment were you receiving for your bowel condition?

**Please check (X) ONE only:**

□ Injectable prescription medicines

□ Prescription medicines taken by mouth

□ Surgery

□ Prescription medicines and surgery

□ No treatment

Overall, do you prefer the drug that you received since you entered this trial to the treatment you received before this clinical trial?

**Please check (X) ONE only:**

□ (1) Yes, I definitely prefer the drug that I am receiving now

□ (2) I have a slight preference for the drug that I am receiving now

□ (3) I have no preference either way

□ (4) I have a slight preference for my previous treatment

□ (5) No, I definitely prefer my previous treatment

**(3) Patient Willingness To Use Drug Again Assessment**

In the future, would you be willing to use the same drug that you have received since you entered this trial for your bowel condition?

**Please check (X) ONE only:**

□ (1) Yes, I would definitely want to use the same drug again

□ (2) I might want to use the same drug again

□ (3) I am not sure

□ (4) I might not want to use the same drug again

□ (5) No, I definitely would not want to use the same drug again
