# Supplementary material for: Randomized trial of tofacitinib in active ulcerative colitis: analysis of efficacy based on patient-reported outcomes
Source: BMC Gastroenterol. 2015 Feb 5;15:14. doi: 10.1186/s12876-015-0239-9 (PMC4323227; doi:10.1186/s12876-015-0239-9)
Supplement: Additional file 2: — Institutional review board that approved the study. Full names of every Institutional review board that approved the study in each center. [file 12876_2015_239_MOESM2_ESM.docx]

## Additional file 2. IBD remission status (endoscopic remission vs IBDQ remission [total score ≥170]).

| **Endoscopic remission, N (%)** | **IBDQ remission (total score ≥170)** | | |
| --- | --- | --- | --- |
|  | **No** | **Yes** | **Total** |
| **No** | 64 (55.2) | 52 (44.8) | 116 (100.0) |
| **Yes** | 9 (30.0) | 21 (70.0) | 30 (100.0) |
| **Total** | 73 (50.0) | 73 (50.0) | 146 (100.0) |

N, number of observations; %, percentage of the total frequency for the row.

IBD, inflammatory bowel disease; IBDQ, Inflammatory Bowel Disease Questionnaire.
