# Supplementary material for: Randomized trial of tofacitinib in active ulcerative colitis: analysis of efficacy based on patient-reported outcomes
Source: BMC Gastroenterol. 2015 Feb 5;15:14. doi: 10.1186/s12876-015-0239-9 (PMC4323227; doi:10.1186/s12876-015-0239-9)
Supplement: Additional file 3: — Inflammatory Bowel Disease Patient-Reported Treatment Impact. Components of the IBD Patient-Reported Treatment Impact questionnaire. Each of the three questions (except the question on previous treatment, which was informational only) was scored on a 5-point scale. [file 12876_2015_239_MOESM3_ESM.pdf]

090177e181c13ab2\Approved\Approved On: 07-Feb-2011 15:21

A4 LIST OF INVESTIGATORS AND CORRESPONDING ETHICS COMMITTEES OR INSTITUTIONAL REVIEW BOARDS

Belgium

Coordinating Investigators:
<None Entered>

Center	Principal Investigator	Co-Investigator(s)	Sub-Investigator(s)	Address(es)	Institutional Review Board or Ethics Committee Address(es)
1029	Dr. Harald Peeters		Dr. Danny De Looze<br>Prof. Martine De Vos	Universitair Ziekenhuis Gent<br>Maag-, darm- en leverziekten<br>De Pintelaan 185<br>Gent, 9000<br>BELGIUM	Universitaire Ziekenhuizen Leuven - Campus Gasthuisberg<br>Commissie voor Medische Ethiek / Klinisch Onderzoek<br>Herestraat 49<br>Leuven, 3000<br>BELGIUM
1030	Prof. Severine A. R. A. Vermeire		Dr. Maja Noman<br>Prof. Paul J. Rutgeerts<br>Prof. Gert A. Van Assche	Universitaire Ziekenhuizen Leuven - Campus Gasthuisberg<br>Gastroenterology<br>Herestraat 49<br>Leuven, 3000<br>BELGIUM	Universitaire Ziekenhuizen Leuven - Campus Gasthuisberg<br>Commissie voor Medische Ethiek / Klinisch Onderzoek<br>Herestraat 49<br>Leuven, 3000<br>BELGIUM
1031	Dr. Joannes Holvoet		Dr. Joris Dutre<br>Dr. Pierre L.A. Gigase<br>Dr. Isabelle Ruytjens	Ziekenhuisnetwerk Antwerpen Middelheim<br>Gastro-enterology<br>Lindendreef 1<br>Antwerpen, 2020<br>BELGIUM	Universitaire Ziekenhuizen Leuven - Campus Gasthuisberg<br>Commissie voor Medische Ethiek / Klinisch Onderzoek<br>Herestraat 49<br>Leuven, 3000<br>BELGIUM

* Did not randomize subjects

090177e181c13ab2\Approved\Approved On: 07-Feb-2011 15:21

Brazil

Coordinating Investigators:
<None Entered>

Center	Principal Investigator	Co-Investigator(s)	Sub-Investigator(s)	Address(es)	Institutional Review Board or Ethics Committee Address(es)
1063	Dr. Luiz Carlos Marquetti		Dr. Fatima Regina Marques Abreu<br>Dr. Freddy Goldberg Eliaschewitz<br>Dr. Carlos Valter Sobrado Jr	CPCLIN - Centro de Pesquisas Clinicas Ltda<br>Rua Goias, 193<br>Higienopolis<br>Sao Paulo, SP 01244-030<br>BRAZIL	Irmandade da Santa Casa de Misericordia de Sao Paulo -ISCMSP<br>Rua Santa Isabel,305<br>4 andar -Santa Cecilia<br>Sao Paulo, SP 01221-010<br>BRAZIL
1064	Dr. Marta Brenner Machado		Dra Luciana Schuck Guedes	Hospital São Lucas da PUCRS<br>Av. Ipiranga, 6690 4 andar<br>Jardim Botanico<br>Porto Alegre, RS 90610-000<br>BRAZIL	Comitê de Ética em Pesquisa da Pontifícia Universidade Católica do Rio Grande do Sul - PUCRS<br>Hospital São Lucas da PUCRS<br>Avenida Ipiranga, 6690 ¿ sala 228/02<br>Jardim Botanico<br>Porto Alegre, RS 90610-000<br>BRAZIL
1074	Dr Flavio Steinwurz		Dr Ricardo L Ganc<br>Dr Karime Lucas<br>Dra Maria Luiza Queiroz Miranda<br>Dr Fernanda Eliza Simoes<br>Dr Andrea Vieira	Hospital Israelita Albert Einstein<br>Centro de Pesquisa Clínica - IIEP<br>Av. Albert Einstein<br>2 Subsolo ¿ Bloco A- Morumbi - 627/701<br>São Paulo, SP 05651-901<br>BRAZIL	Comite de Etica em Pesquisa do Hospital Israelita Albert Einstein<br>Avenida Albert Einstein, 627 / 701 - Bloco A - 2o Subsolo<br>Morumbi<br>São Paulo, São Paulo 05651-901<br>BRAZIL

* Did not randomize subjects

090177e181c13ab2\Approved\Approved On: 07-Feb-2011 15:21

Chile

Coordinating Investigators:
<None Entered>

Center	Principal Investigator	Co-Investigator(s)	Sub-Investigator(s)	Address(es)	Institutional Review Board or Ethics Committee Address(es)
1081 *	Sergio de la Barra		Roberto Nazal	Complejo Hospitalario San Jose<br>Unidad de Gastroenterologia-Endoscopia<br>Profesor Alberto Zanartu 1085<br>Independencia, Santiago 8380418<br>CHILE	Comite de Etica de Investigacion<br>Servicio de Salud Metropolitano Norte<br>Calle San Jose # 1053<br>Independencia, RM<br>CHILE
1083	Carlos Bustos		Roberto Leon Sade	Hospital Dr. Gustavo Fricke<br>Alvarez 1532<br>Vina del Mar, 2570017<br>CHILE	Comite Etico Cientifico del Servicio de Salud Vina-Quillota<br>Calle Limache 1307<br>Esquina Penablanca, Piso 2<br>Vina del Mar, V Region<br>CHILE
1085	Maria Ester Bufadel		Carolina Figueroa<br>Carolina Pizarro<br>Jorge Valenzuela	Hospital Clinico Universidad de Chile<br>Servicio Gastroenterologia, 3er Piso, Sector D<br>Santos Dumont 999<br>Independencia, Santiago RM 8380456<br>CHILE	Comite de Etica de Investigacion<br>Servicio de Salud Metropolitano Norte<br>Calle San Jose # 1053<br>Independencia, RM<br>CHILE<br><br>Comite de Etica, Hospital Clinico Universidad de Chile<br>Santos Dummont 999, 6to Piso, Sector E<br>Independencia, Santiago, RM<br>CHILE

* Did not randomize subjects

090177e181c13ab2\Approved\Approved On: 07-Feb-2011 15:21

Czech Republic

Coordinating Investigators:
<None Entered>

Center	Principal Investigator	Co-Investigator(s)	Sub-Investigator(s)	Address(es)	Institutional Review Board or Ethics Committee Address(es)
1027	Dr. Jiri Stehlik		Dr. Jiri Lastuvka<br>Dr. Michal Tichy	Masarykova nemocnice<br>Gastroenterologie<br>Socialni pece 3316/12A<br>Usti n. Labem, 401 13<br>CZECH REPUBLIC<br><br>Masarykova nemocnice<br>Socialni pece 3316/12A<br>Usti nad Labem, 401 13<br>CZECH REPUBLIC	Eticka Komise FN Hradec Kralove<br>Hradecka ulice 1172<br>Hradec Kralove, 500 03<br>CZECH REPUBLIC<br><br>Eticka komise Masarykova nemocnice<br>Prispevkova organizace<br>Socialni pece 3316/12A<br>Usti nad Labem, 401 13<br>CZECH REPUBLIC
1071	Dr. Michal Konecny			Fakultni nemocnice Olomouc<br>I. P. Pavlova 6<br>Olomouc, 775 20<br>CZECH REPUBLIC	Eticka Komise FN Hradec Kralove<br>Hradecka ulice 1172<br>Hradec Kralove 3, 500 03<br>CZECH REPUBLIC<br><br>Multicenticka eticka komise Fakultni nemocnice Olomouc<br>I.P.Pavlova 6<br>Olomouc, 77520<br>CZECH REPUBLIC
1084	Dr. Tomas Vanasek		Dr. Pavla Loudova<br>Dr. Miroslava Volfova	Hepato-Gastroenterologie HK, s.r.o.<br>Poliklinika III<br>Trida Edvarda Benese 1549<br>Hradec Kralove, 50012<br>CZECH REPUBLIC	Eticka Komise FN Hradec Kralove<br>Hradecka ulice 1172<br>Hradec Kralove, 500 03<br>CZECH REPUBLIC

* Did not randomize subjects

Denmark

Coordinating Investigators:
<None Entered>

Center	Principal Investigator	Co-Investigator(s)	Sub-Investigator(s)	Address(es)	Institutional Review Board or Ethics Committee Address(es)
1023	Dr. Jens Frederik Dahlerup		Dr. Joergen Steen Agnholt<br>Dr. Lisbet Ambrosius Christensen	Aarhus Universitetshospital, Aarhus Sygehus<br>Medicinsk Hepato-gastroenterologisk Afdeling V<br>Noerrebrogade 44, Bygning 7<br>Aarhus C, 8000<br>DENMARK	De Videnskabsetiske Komitéer for Region Midtjylland<br>Sundhedssekretariatet<br>P.O. Box 21<br>Skottenborg 26<br>Viborg, 8800<br>DENMARK
1024	Dr. med. Jan Fallingborg		Dr. Bent A. Jacobsen<br>Dr. Lone Larsen<br>Dr. Henrik Hoejgaard Rasmussen	Aalborg Sygehus<br>Medicinsk Gastroenterologisk afdeling - Medicinerhuset<br>Moelleparkvej 4, postboks 561<br>Aalborg, 9100<br>DENMARK	De Videnskabsetiske Komitéer for Region Midtjylland<br>Sundhedssekretariatet<br>P.O. Box 21<br>Skottenborg 26<br>Viborg, 8800<br>DENMARK

090177e181c13ab2\Approved\Approved On: 07-Feb-2011 15:21

France

Coordinating Investigators:
<None Entered>

Center	Principal Investigator	Co-Investigator(s)	Sub-Investigator(s)	Address(es)	Institutional Review Board or Ethics Committee Address(es)
1057	Prof. Jean Frederic Colombel		Dr. Julien Branche<br>Dr. Antoine Cortot<br>Prof. Pierre Desreumaux<br>Peggy Fournier<br>Dr. Elsa Jozefowicz<br>Gwenola Vernier<br>Dr. Faustine Wartel	Hopital Huriez, CHRU de Lille<br>Service d'Hepato gastro-enterologie<br>1 place de Verdun<br>Lille Cedex, FRANCE 59037<br>FRANCE	Comité de protection des personnes <Nord Ouest IV><br>CPP Nord Ouest IV<br>Service de Pharmacologie<br>Faculté de Médecine - Pôle Recherche<br>1, Place de Verdun<br>LILLE cedex, 59045<br>FRANCE
1059	Pr. Jean-Charles Grimaud		Dr. Ariadne Desjeux<br>Dr. Catherine Lesavre<br>Dr. Annick Pelletier	Hôpital Nord<br>Service d'Hépato-Gastro-Enterologie<br>Chemin Bourrely<br>Marseille cedex 20, 13915<br>FRANCE	Comité de protection des personnes <Nord Ouest IV><br>CPP Nord Ouest IV<br>Service de Pharmacologie<br>Faculté de Médecine - Pôle Recherche<br>1, Place de Verdun<br>LILLE cedex, 59045<br>FRANCE
1061	Pr. Frank Zerbib		Dr. Pauline Roumeguere	Hopital Saint-Andre<br>Service d'Hepatogastoenterologie<br>Bordeaux cedex, 33075<br>FRANCE	Comité de protection des personnes <Nord Ouest IV><br>CPP Nord Ouest IV<br>Service de Pharmacologie<br>Faculté de Médecine - Pôle Recherche<br>1, Place de Verdun<br>LILLE cedex, 59045<br>FRANCE

* Did not randomize subjects

090177e181c13ab2\Approved\Approved On: 07-Feb-2011 15:21

**Center**	**Principal Investigator**	**Co-Investigator(s)**	**Sub-Investigator(s)**	**Address(es)**	**Institutional Review Board or Ethics Committee Address(es)**
1068	Dr. Arnaud Bourreille<br>Prof. Jean-paul Galmiche (Previous PI)		Dr. Mathurin Flamand	CHU Hôtel-Dieu<br>Service Hepato-gastroentérologie<br>1 Place Alexis Ricordeau<br>Nantes CEDEX 1, 44093<br>FRANCE	Comité de protection des personnes <Nord Ouest IV><br>CPP Nord Ouest IV<br>Service de Pharmacologie<br>Faculté de Médecine - Pôle Recherche<br>1, Place de Verdun<br>LILLE cedex, 59045<br>FRANCE

090177e181c13ab2\Approved\Approved On: 07-Feb-2011 15:21

* Did not randomize subjects

Hungary

Coordinating Investigators:
<None Entered>

Center	Principal Investigator	Co-Investigator(s)	Sub-Investigator(s)	Address(es)	Institutional Review Board or Ethics Committee Address(es)
1032	Dr. Marta Varga		Dr. Klara Csefko<br>Dr. Ildiko Soos<br>Dr. Ildiko Soos<br>Dr. Mariann Szabo	Dr. Rethy Pal Korhaz es Rendelointezet/III. sz. Belgyogyaszati Osztaly<br>Gyulai ut 18.<br>Bekescsaba, 5600<br>HUNGARY	Egeszsegugyi Tudomanyos Tanacs Klinikai Farmakologiai Etikai Bizottsaga<br>Arany J. u. 6-8.<br>Budapest, H-1051<br>HUNGARY
1033	Prof. Margit Zeher		Dr. Zsolt Barta<br>Dr. Eva Zold	Debreceni Egyetem Orvos- es Egeszsegtudo-manyi Centrum/III. sz. Belgyogyaszati Klinika<br>Moricz Zsigmond krt. 22.<br>Debrecen, 4004<br>HUNGARY	Egeszsegugyi Tudomanyos Tanacs Klinikai Farmakologiai Etikai Bizottsaga<br>Arany J. u. 6-8.<br>Budapest, H-1051<br>HUNGARY
1034	Dr Janos Novak		Dr. Ilona Fazekas<br>Dr. Zoltan Gurzo<br>Dr. Sandor Ilyes<br>Dr. Laszlo Szalai	Bekes Megyei Kepviselo-testulet Pandy Kalman Korhaza<br>Semmelweis u. 1.<br>Gyula, 5701<br>HUNGARY	Egeszsegugyi Tudomanyos Tanacs Klinikai Farmakologiai Etikai Bizottsaga<br>Arany J. u. 6-8.<br>Budapest, H-1051<br>HUNGARY
1035	Prof. Laszlo Ujszaszy		Dr. Gabor Horvath<br>Dr. Janos Theisz	Miskolc Megyei Jogu Varos onkormanyzata Semmelweis Korhaz-Rendelointezet és Egyetemi Oktato Korhaz<br>Csabai kapu 9-11.<br>Miskolc, 3529<br>HUNGARY	Egeszsegugyi Tudomanyos Tanacs Klinikai Farmakologiai Etikai Bizottsaga<br>Arany J. u. 6-8.<br>Budapest, H-1051<br>HUNGARY

* Did not randomize subjects

090177e181c13ab2\Approved\Approved On: 07-Feb-2011 15:21

090177e181c13ab2\Approved\Approved On: 07-Feb-2011 15:21

Center	Principal Investigator	Co-Investigator(s)	Sub-Investigator(s)	Address(es)	Institutional Review Board or Ethics Committee Address(es)
1036	Prof. Bela Hunyady		Dr. Andras Deak<br>Dr. Eva Graffits<br>Dr. Attila Haragh<br>Dr. Zoltan Kovacs<br>Dr. Klara Kubinyi<br>Dr. Csaba Sulle	Kaposi Mor Oktato Korhaz /<br>Belgyogyaszati osztaly<br>Tallian Gy. u. 20-32.<br>Kaposvar, 7400<br>HUNGARY	Egeszsegugyi Tudomanyos Tanacs<br>Klinikai Farmakologiai Etikai<br>Bizottsaga<br>Arany J. u. 6-8.<br>Budapest, H-1051<br>HUNGARY
1037	Dr. Tamas Molnar		Dr. Klaudia Farkas<br>Dr. Ferenc Nagy<br>Dr. Zoltan Szepes<br>Dr. Tibor Wittmann	Szegedi Tudomanyegyetem<br>altalanos Orvostudomanyi Kar / I.<br>sz. Belgyogyaszati Klinika<br>Tisza L. korut 107<br>Szeged, 6720<br>HUNGARY	Egeszsegugyi Tudomanyos Tanacs<br>Klinikai Farmakologiai Etikai<br>Bizottsaga<br>Arany J. u. 6-8.<br>Budapest, H-1051<br>HUNGARY
1038	Dr. Agnes Salamon		Dr. Ferenc Felfoldi<br>Dr. Iren Matai<br>Dr. Judit Santa<br>Dr. Beatrix Tam<br>Dr. Gyula Toth<br>Dr. Edit Vadasz	Clinfan Kft.<br>Beri Balogh Adam u. 5-7.<br>Szekszard, 7100<br>HUNGARY	Egeszsegugyi Tudomanyos Tanacs<br>Klinikai Farmakologiai Etikai<br>Bizottsaga<br>Arany J. u. 6-8.<br>Budapest, H-1051<br>HUNGARY
1039	Prof. Istvan Racz		Dr. Mihaly Csondes<br>Dr Saleh Hussam<br>Dr. Tibor Karasz<br>Dr. Andrea Szabo	Petz Aladar Megyei Oktato<br>Korhaz / I Belgyogyaszat<br>Vasvari Pal u. 2-4.<br>Gyor, 9023<br>HUNGARY	Egeszsegugyi Tudomanyos Tanacs<br>Klinikai Farmakologiai Etikai<br>Bizottsaga<br>Arany J. u. 6-8.<br>Budapest, H-1051<br>HUNGARY
1052	Dr. Gyula Pecsi		Dr. Zsuzsanna Fuzi<br>Dr. Tibor Szabo<br>Dr. Lajos Toth	Karolina Korhaz Rendelointezet,<br>Belgyogyaszat<br>Regi Vamhaz ter 2-4.<br>Mosonmagyarovar, 9200<br>HUNGARY	Egeszsegugyi Tudomanyos Tanacs<br>Klinikai Farmakologiai Etikai<br>Bizottsaga<br>Arany J. u. 6-8.<br>Budapest, H-1051<br>HUNGARY

* Did not randomize subjects

090177e181c13ab2\Approved\Approved On: 07-Feb-2011 15:21

Center	Principal Investigator	Co-Investigator(s)	Sub-Investigator(s)	Address(es)	Institutional Review Board or Ethics Committee Address(es)
1053	Dr. Gyorgy Szekely		Dr. Bela Boka<br>Dr. Gabor Sipos<br>Dr. Gabor Tamas Toth	Fovarosi Onkormanyzat Szent Janos Korhaz es Rendelointezet, I. Belgyogyaszat es Gastroenterologia<br>Dios arok 1-3.<br>Budapest, 1125<br>HUNGARY	Egeszsegugyi Tudomanyos Tanacs Klinikai Farmakologiai Etikai Bizottsaga<br>Arany J. u. 6-8.<br>Budapest, H-1051<br>HUNGARY
1054	Dr. Robert Schnabel		Dr. Sandor Molnar<br>Dr. Valeria Sipos<br>Dr. Mariann Vadnai<br>Dr. Patricia Varga	Fovarosi Onkormanyzat Nyiro Gyula Korhaz, 2. sz. Belgyogyaszati Osztaly<br>Lehel u. 59.<br>Budapest, 1135<br>HUNGARY	Egeszsegugyi Tudomanyos Tanacs Klinikai Farmakologiai Etikai Bizottsaga<br>Arany J. u. 6-8.<br>Budapest, H-1051<br>HUNGARY
1055	Dr. Barnabas Bod		Dr. Aranka Kovacs	Dr. Bugyi Istvan Korhaz, Gasztroenterologia<br>Sima F. u. 44-58.<br>Szentes, 6600<br>HUNGARY	Egeszsegugyi Tudomanyos Tanacs Klinikai Farmakologiai Etikai Bizottsaga<br>Arany J. u. 6-8.<br>Budapest, H-1051<br>HUNGARY

* Did not randomize subjects

090177e181c13ab2\Approved\Approved On: 07-Feb-2011 15:21

Israel

Coordinating Investigators:
<None Entered>

Center	Principal Investigator	Co-Investigator(s)	Sub-Investigator(s)	Address(es)	Institutional Review Board or Ethics Committee Address(es)
1073	Prof. Yaron Niv		Dr. Zaza Beniashvili<br>Dr. Ram Menashe Dickman<br>Dr. Gerald Martin Fraser<br>Dr. Moshe Furman<br>Dr. Eyal Gal<br>Dr. Zohar Levy<br>Dr. Lev Lichtenstein<br>Dr. Boris Sapoznikov<br>Dr. Alexander Vilkin	Rabin Medical Centre Beilinson Campus, Gastroenterology Department<br>Petah-tikva, 49100<br>ISRAEL	Helsinki Committee,Rabin Medical Centre<br>Rabin Medical Centre Beilinson Campus<br>Petah-Tikva, 49100<br>ISRAEL
1077	Dr. Sigal Fishman		Dr. Iris Dotan<br>Dr. Roman Grenshpon<br>Dr. Revital Kariv<br>Dr. Nitsan Maharshak<br>Dr. Jorge Pfeffer<br>Dr. Yulia Ron<br>Dr. Erez Scapa<br>Dr. Yami Shapira	Department of Gastroenterology & Hepatology, Tel Aviv Sourasky Medical Center<br>6 Weizman St.<br>Tel Aviv, 64239<br>ISRAEL	Helsinki Committee<br>The Helsinki committee, Tel Aviv Sourasky Medical Center<br>6 Weizman Street<br>Tel Aviv, 64239<br>ISRAEL
1080 *	Prof. Abraham Eliakim		Dr. Irit Chermesh<br>Dr. Yehuda Chowers<br>Dr. Amir Klein<br>Dr. Itay Maza<br>Dr. Kamel Yassin	Gastroenterology Insitute, Rambam Medical Center<br>PO Box 9602<br>Haifa, 31096<br>ISRAEL	Rambam Medical Center Helsinki Committee<br>Rambam Medical Center<br>PO Box 9602<br>Haifa, 31096<br>ISRAEL

* Did not randomize subjects

Italy

Coordinating Investigators:
<None Entered>

Center	Principal Investigator	Co-Investigator(s)	Sub-Investigator(s)	Address(es)	Institutional Review Board or Ethics Committee Address(es)
1004	Prof. Paolo Gionchetti		Dr. Ramona Brugnera<br>Prof. Fernando Rizzello<br>Dr. Giulia Straforini<br>Dr. Rosy Tambasco	Dipartimento di Medicina Clinica<br>SSD Malattie Infiammatorie<br>Croniche Intestinali<br>Azienda Ospedaliero<br>Universitaria di Bologna<br>Policlinico Sant'Orsola Malpighi<br>Via Massarenti, 9<br>Bologna, 40138<br>ITALY	Comitato Etico Indipendente<br>dell'Azienda Ospedaliero<br>Universitaria, Policlinico Sant'<br>Orsola Malpighi di Bologna,<br>Padiglione 3<br>Via Albertoni, 15<br>Bologna, 40138<br>ITALY
1006 *	Anna Kohn<br>Prof Cosimo Prantera<br>(Previous PI)		Dr. Patrizia Meddi<br>Rita Monterubbianesi<br>Dr. Maria Lia Scribano<br>Dr. Antonio Tesi	Azienda Ospedaliera Sanitaria<br>San Camillo Forlanini<br>U.O.C. di Gastroenterologia,<br>Padiglione Bassi, II piano<br>Via Gianicolense, 87<br>Roma, 00152<br>ITALY	Comitato Etico dell'Azienda<br>Ospedaliera San Camillo Forlanini di<br>Roma<br>Circonvallazione Gianicolense, 87<br>Roma, 00152<br>ITALY

090177e181c13ab2\Approved\Approved On: 07-Feb-2011 15:21

* Did not randomize subjects

Mexico

Coordinating Investigators:
<None Entered>

Center	Principal Investigator	Co-Investigator(s)	Sub-Investigator(s)	Address(es)	Institutional Review Board or Ethics Committee Address(es)
1065	Jesus K Yamamoto-Furusho		Dr. Enrique Coss-Adame<br>Claudia Herrera-de Guise	Instituto Nacional de Ciencias Medicas y Nutricion Salvador Zubiran<br>Departamento de Gastroenterologia 2do Piso<br>Vasco de Quiroga 15<br>Col Seccion XVI<br>Tlalpan, Mexico DF 14000<br>MEXICO	Instituto Nacional de Ciencias Medicas y Nutricion Salvador Zubiran<br>Comite Institucional de Investigacion Biomedica en Humanos<br>VASCO DE QUIROGA 15<br>COL SECCION XVI TLALPAN<br>Mexico, DF 14000<br>MEXICO
1066	Pedro Lopez-Fournier		Dr. Reyna Manuela Bustamante-Gonzalez<br>Dr. Luis Kenji Carmona-Furusho<br>Dr. Norma Alicia Martinez-Trejo<br>Dr. Reyna Mendez-del-Monte	Clinicos Asociados BOCM, S.C<br>Victor Hugo 191-BIS Altos<br>Col Portales<br>Delegacion Benito Juarez, Mexico DF 03300<br>MEXICO	Comite Bioetico para la Investigacion Clinica S.C.<br>Puebla 422<br>Despacho 4<br>Col. Roma Sur<br>MEXICO, DISTRITO FEDERAL 06700<br>MEXICO

* Did not randomize subjects

090177e181c13ab2\Approved\Approved On: 07-Feb-2011 15:21

Netherlands

Coordinating Investigators:
<None Entered>

Center	Principal Investigator	Co-Investigator(s)	Sub-Investigator(s)	Address(es)	Institutional Review Board or Ethics Committee Address(es)
1018	Dr. Ad A. van Bodegraven gastro-enterologist		Dr. G. Bouma<br>Dr. D. P. van Asseldonk	VU Medisch Centrum<br>De Boelelaan 1118<br>Amsterdam, NH 1081 HV<br>NETHERLANDS	METC VU Medisch Centrum<br>PK 6 Z 202<br>Postbus 7075<br>Amsterdam, 1007 MB<br>NETHERLANDS

090177e181c13ab2\Approved\Approved On: 07-Feb-2011 15:21

* Did not randomize subjects

090177e181c13ab2\Approved\Approved On: 07-Feb-2011 15:21

Poland

Coordinating Investigators:
<None Entered>

Center	Principal Investigator	Co-Investigator(s)	Sub-Investigator(s)	Address(es)	Institutional Review Board or Ethics Committee Address(es)
1010	Prof. Leszek Paradowski		Dr. Jadwiga Lapinska<br>Dr. Barbara Wozniak-Stolarska	Klinika Gastroenterologii i Hepatologii<br>Akademicki Szpital Kliniczny im. Jana Mikulicza-Radeckiego we Wroclawiu<br>ul. Borowska 213<br>Wroclaw, 50-556<br>POLAND	Komisja Bioetyki Uniwersytetu Medycznego w Lodzi<br>Al. Kosciuszki 4<br>Lodz, 90-419<br>POLAND
1013	Prof. Ewa Malecka-Panas		Dr. Anita Gasiorowska<br>Dr. Justyna Kotynia<br>Dr. Renata Talar-Wojnarowska	Oddzial Kliniczny Gastroenterologii Ogolnej i Onkologicznej<br>SPZOZ Uniwersytecki Szpital Kliniczny nr 1 im. Norberta Barlickiego Uniwersytetu Medycznego w Lodzi<br>ul.Kopcinskiego 22<br>Lodz, 90-153<br>POLAND	Komisja Bioetyki Uniwersytetu Medycznego w Lodzi<br>Al. Kosciuszki 4<br>Lodz, 90-419<br>POLAND
1049	Prof. Maciej Swiatkowski		Dr. Malgorzata Brymora<br>Dr. Maria Klopocka<br>Dr. Marcin Manerowski<br>Dr. Agnieszka Meder	Oddzial Kliniczny Gastroenterologii, Angiologii i Chorob Wewnetrznych<br>Szpital Uniwersytecki nr 2 im. Dr Jana Biziela w Bydgoszczy<br>ul. Ujejskiego 75<br>Bydgoszcz, 85-168<br>POLAND	Komisja Bioetyki Uniwersytetu Medycznego w Lodzi<br>Al. Kosciuszki 4<br>Lodz, 90-419<br>POLAND

* Did not randomize subjects

Slovakia

Coordinating Investigators:
<None Entered>

Center	Principal Investigator	Co-Investigator(s)	Sub-Investigator(s)	Address(es)	Institutional Review Board or Ethics Committee Address(es)
1043	Dr. Ladislav Kuzela		Dr. Peter Paulen	Lama Medical Care s.r.o<br>Gastroentero-hepatologicke centrum Thalion<br>Mytna 5<br>Bratislava, 811 07<br>SLOVAKIA	Eticka komisia<br>Univerzitna nemocnica Bratislava<br>Limbova 5<br>Bratislava, SLOVAKIA 833 05<br>SLOVAKIA<br><br>Eticka komisia Bratislavskeho samospravneho kraja<br>Eticka komisia Bratislavskeho samospravneho kraja<br>Sabinovska 16<br>P.O. Box 106<br>Bratislava, 820 05<br>SLOVAKIA
1044	Dr. Boris Baricky			Medicinske centrum Nitra spol. s r.o.<br>Gastroenterologicka ambulancia<br>Fatranska 12<br>Nitra, 94901<br>SLOVAKIA	Eticka komisia<br>Univerzitna nemocnica Bratislava<br>Limbova 5<br>Bratislava, SLOVAKIA 833 05<br>SLOVAKIA<br><br>Eticka komisia Nitrianskeho samospravneho kraja<br>Stefanikova tr. 69<br>Nitra, 949 01<br>SLOVAKIA

090177e181c13ab2\Approved\Approved On: 07-Feb-2011 15:21

Center	Principal Investigator	Co-Investigator(s)	Sub-Investigator(s)	Address(es)	Institutional Review Board or Ethics Committee Address(es)
1079	Tibor Hlavaty		Tomas Koller<br>Jozef Toth	Gastroenterologicke a hepatologicke oddelenie, V. interna klinika LFUK a UN Bratislava, Ruzinov<br>Ruzinovska 6<br>Bratislava, SLOVAKIA 826 06<br>SLOVAKIA	Eticka komisia<br>Univerzitna nemocnica Bratislava<br>Limbova 5<br>Bratislava, SLOVAKIA 833 05<br>SLOVAKIA<br><br>Eticka komisia<br>Univerzitna nemocnica Bratislava<br>Ruzinovska 6<br>Bratislava, 826 06<br>SLOVAKIA

090177e181c13ab2\Approved\Approved On: 07-Feb-2011 15:21

* Did not randomize subjects

090177e181c13ab2\Approved\Approved On: 07-Feb-2011 15:21

South Africa

Coordinating Investigators:
<None Entered>

Center	Principal Investigator	Co-Investigator(s)	Sub-Investigator(s)	Address(es)	Institutional Review Board or Ethics Committee Address(es)
1047	Dr John P. Wright		Dr. Abdul Karriem Cariem	Kingsbury Hospital<br>301 Fairfields Suite<br>Wildernis Road<br>Claremont, Western Cape 7708<br>SOUTH AFRICA	South African Medical Association<br>Research Ethics Committee<br>Block F, Castle Walk, Corporate Park<br>Nossob Street<br>Erasmuskloof Ext 3<br>Pretoria, 0153<br>SOUTH AFRICA
1048	Dr Frederik Cornelius Kruger		Dr Jane Elizabeth Christie Botha	Durbanville Medi-clinic<br>Room 106<br>Wellington Str<br>Durbanville, Western Cape 7550<br>SOUTH AFRICA	South African Medical Association<br>Research Ethics Committee<br>Block E Castle Walk Corporate Park<br>Nossob Street Erasmuskloof Ext 3<br>Pretoria, 0153<br>SOUTH AFRICA
1050 *	Herbert R. Schneider		Dr. Brendan Dirk Bebington	Milpark Clinic<br>Room 202 Second Floor<br>9 Guild Road<br>Parktown<br>Johannesburg, Gauteng 2193<br>SOUTH AFRICA	South African Medical Association<br>Research Ethics Committee<br>Block E Castle Walk Corporate Park<br>Nossob Street Erasmuskloof Ext 3<br>Pretoria, 0153<br>SOUTH AFRICA

* Did not randomize subjects

090177e181c13ab2\Approved\Approved On: 07-Feb-2011 15:21

Center	Principal Investigator	Co-Investigator(s)	Sub-Investigator(s)	Address(es)	Institutional Review Board or Ethics Committee Address(es)
1062 *	Dr. Suleman Abdul Moola		Ismail Aboobaker Abdullah<br>Dr Sarah Mohammed Moola<br>Dr Anoob Ramdayal Seebaran	St Augustine Medical Centre 2<br>107 Chelsmford Road<br>Berea<br>Durban, Kwa-Zulu Natal 4001<br>SOUTH AFRICA<br><br>St Augustine Medical Centre 2<br>107 JB Marks Road<br>Berea<br>Durban, Kwa-Zulu Natal 4001<br>SOUTH AFRICA	South African Medical Association<br>Research Ethics Committee<br>Block F, Castle Walk, Corporate Park<br>Nossob Street<br>Erasmuskloof Ext 3<br>Pretoria, 0153<br>SOUTH AFRICA
1069	Dr. Nazimuddin Aboo		Dr. Harshadkumar Ishwarial Rajput	Parklands Medical Centre<br>Suite 1A<br>75 Hopelands Road<br>Overport, Durban 4091<br>SOUTH AFRICA	South African Medical Association<br>Research Ethics Committee<br>Block E Castle Walk Corporate Park<br>Nossob Street Erasmuskloof Ext 3<br>Pretoria, 0153<br>SOUTH AFRICA

* Did not randomize subjects

090177e181c13ab2\Approved\Approved On: 07-Feb-2011 15:21

Spain

Coordinating Investigators:
<None Entered>

Center	Principal Investigator	Co-Investigator(s)	Sub-Investigator(s)	Address(es)	Institutional Review Board or Ethics Committee Address(es)
1020	Maria Isabel Vera Mendoza		Juan De la Revilla<br>Virginia Matallana Royo<br>Maria Belen Ruiz Antoran<br>Arantxa Sancho Lopez	HOSPITAL UNIVERSITARIO PUERTA DE HIERRO MAJADAHONDA<br>SERVICIO DE DIGESTIVO<br>C/ MANUEL DE FALLA 1<br>MAJADAHONDA, MADRID 28222<br>SPAIN	Hospital Clinico San Carlos<br>ETHICS COMMITTEE OF CLINICAL INVESTIGATION AREA 7<br>C/ PROFESOR MARTIN LAGOS, S/N<br>MADRID, MADRID 28040<br>SPAIN
1021	Julian Panes Diaz		Montserrat Aceituno<br>Orlando Garcia Bosch<br>Ingrid Ordas Jimenez<br>ELENA RICART<br>Miguel Sans Cuffi	HOSPITAL CLINIC I PROVINCIAL DE BARCELONA<br>SERVICIO DE DIGESTIVO<br>C/ VILLARROEL 170<br>BARCELONA, BARCELONA 08036<br>SPAIN	Hospital Clinico San Carlos<br>ETHICS COMMITTEE OF CLINICAL INVESTIGATION AREA 7<br>C/ PROFESOR MARTIN LAGOS, S/N<br>MADRID, MADRID 28040<br>SPAIN
1022	Jordi Guardiola		Ana Berrozpe Lopez<br>Josep Maria Botargues<br>Marcela Manriquez<br>Mireia Peñalva Peñas<br>Francisco Rodriguez<br>CLARA MARIA ROSSO FERNANDEZ<br>Antonio Soriano Izquierdo	HOSPITAL UNIVERSITARI DE BELLVITGE<br>SERVICIO DE DIGESTIVO<br>C/ FEIXA LLARGA, S/N<br>L'HOSPITALET DE LLOBREGAT, BARCELONA 08907<br>SPAIN	Hospital Clinico San Carlos<br>ETHICS COMMITTEE OF CLINICAL INVESTIGATION AREA 7<br>C/ PROFESOR MARTIN LAGOS, S/N<br>MADRID, MADRID 28040<br>SPAIN

* Did not randomize subjects

Sweden

Coordinating Investigators:
<None Entered>

Center	Principal Investigator	Co-Investigator(s)	Sub-Investigator(s)	Address(es)	Institutional Review Board or Ethics Committee Address(es)
1067	Dr. Ake Danielsson		Pontus Karling<br>Olé Suhr<br>Marten Werner	Norrlands Universitetssjukhus,<br>Medicinkliniken<br>Umea, 901 85<br>SWEDEN	Regionala etikprovningsnamnden i Umea<br>Samverkanshuset, Universitetsomradet<br>Umea, 901 87<br>SWEDEN
1070	Kjell-Ake Jonsson		Henrik Simán<br>Adam Witek	Centrallasarettet Vaxjo,<br>Medicinska kliniken<br>Vaxjo, 351 85<br>SWEDEN	Regionala etikprovningsnamnden i Umea<br>Samverkanshuset, Universitetsomradet<br>Umea, 901 87<br>SWEDEN

090177e181c13ab2\Approved\Approved On: 07-Feb-2011 15:21

* Did not randomize subjects

090177e181c13ab2\Approved\Approved On: 07-Feb-2011 15:21

United Kingdom

Coordinating Investigators:
<None Entered>

Center	Principal Investigator	Co-Investigator(s)	Sub-Investigator(s)	Address(es)	Institutional Review Board or Ethics Committee Address(es)
1009 *	Prof. Christopher Probert		Dr. Tom Julian Creed<br>Dr. John Edmund Smithson	Department of Gastroenterology<br>Bristol Royal Infirmary<br>Marlborough Street<br>Bristol, BS2 8HW<br>UNITED KINGDOM	Oxfordshire REC C<br>National Research Ethics Service<br>Ted Co Business Centre<br>Unit 002<br>Rolling Mill Road<br>Jarrow, Tyneside NE32 3DT<br>UNITED KINGDOM<br><br>Oxfordshire Research Ethics Committee C<br>2nd Floor, Astral House<br>Chaucer Business Park<br>Granville Way<br>Bicester, OX26 4JT<br>UNITED KINGDOM
1040	Allan John Morris		Dr Daniel R. Gaya<br>Dr Jack Westwood Winter	Clinical Research Facility<br>Tennent Institute<br>38 Church Street<br>Western Infirmary<br>Glasgow, G11 6NT<br>UNITED KINGDOM<br><br>Ward 8/9<br>Glasgow Royal Infirmary<br>82/84 Castle Street<br>Glasgow, G4 0SS<br>UNITED KINGDOM	Oxfordshire REC C<br>National Research Ethics Service<br>2nd Floor, Astral House<br>Chaucer Business Park, Granville Way<br>Bicester, OX26 4JT<br>UNITED KINGDOM<br><br>Ted Co Business Centre<br>Unit 002<br>Rolling Mill Road<br>Jarrow<br>Tyneside, NE32 3DT<br>UNITED KINGDOM

* Did not randomize subjects

Center	Principal Investigator	Co-Investigator(s)	Sub-Investigator(s)	Address(es)	Institutional Review Board or Ethics Committee Address(es)
1051	Dr. Simon Scott Campbell		Dr. Alistair Makin<br>Dr. Satheesh Nair<br>Dr Robert Paul Willert	Central Manchester University Hospitals Foundation Trust, Department of Gastroenterology<br>Oxford Road<br>Manchester, M13 9WL<br>UNITED KINGDOM	Oxfordshire REC C<br>National Research Ethics Service<br>2nd Floor, Astral House<br>Chaucer Business Park, Granville Way<br>Bicester, OX26 4JT<br>UNITED KINGDOM<br><br>Oxfordshire REC C<br>National Research Ethics Service<br>Ted Co Business Centre<br>Unit 002<br>Rolling Mill Road<br>Jarrow, Tyneside NE32 3DT<br>UNITED KINGDOM

090177e181c13ab2\Approved\Approved On: 07-Feb-2011 15:21

* Did not randomize subjects
