# Supplementary material for: Randomized trial of tofacitinib in active ulcerative colitis: analysis of efficacy based on patient-reported outcomes
Source: BMC Gastroenterol. 2015 Feb 5;15:14. doi: 10.1186/s12876-015-0239-9 (PMC4323227; doi:10.1186/s12876-015-0239-9)
Supplement: Additional file 4: — Patient disposition. patient disposition in the study. [file 12876_2015_239_MOESM4_ESM.pdf]

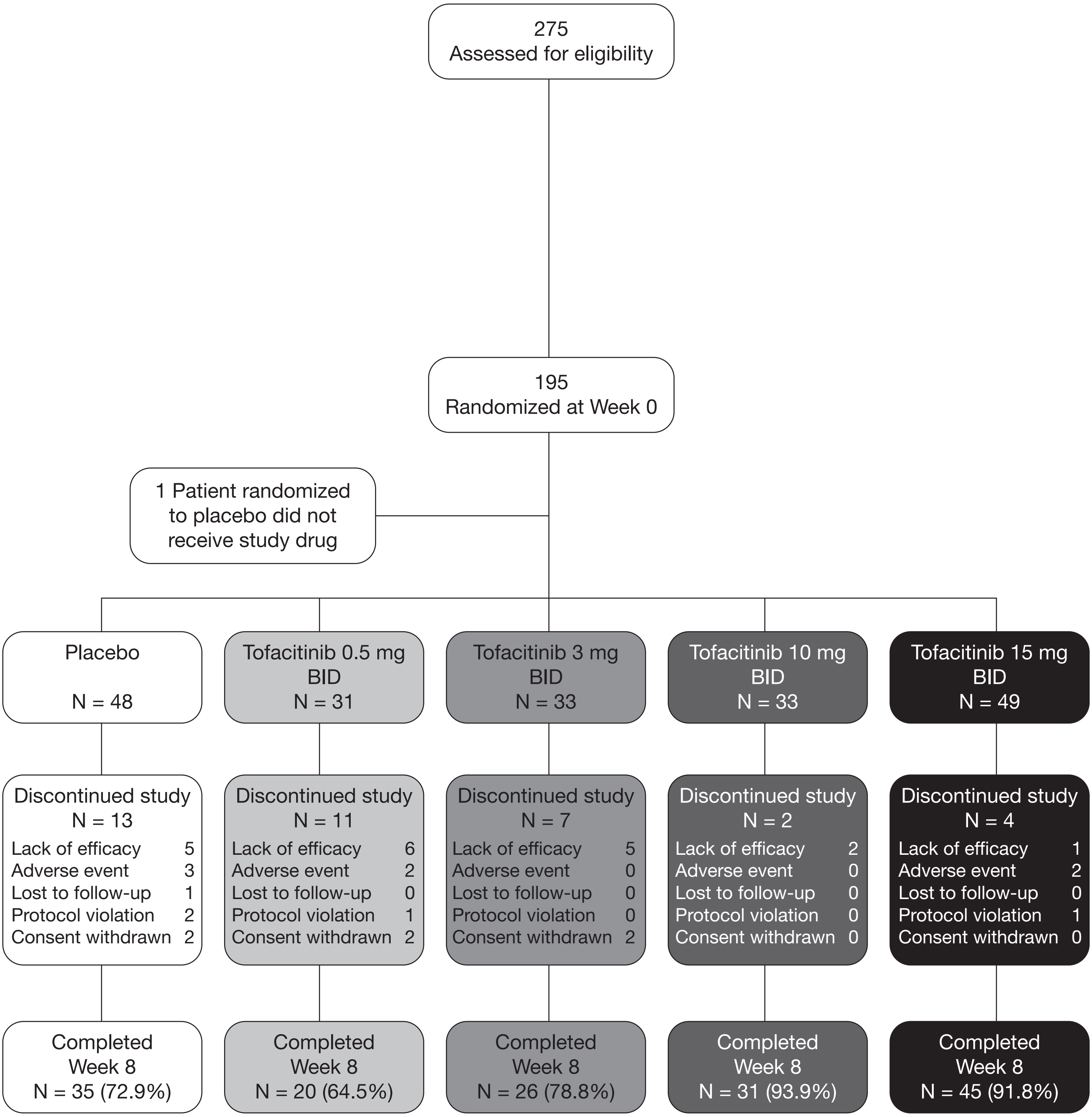
275
Assessed for eligibility
195
Randomized at Week 0
1 Patient randomized to placebo did not receive study drug
Placebo
N = 48
Tofacitinib 0.5 mg BID
N = 31
Tofacitinib 3 mg BID
N = 33
Tofacitinib 10 mg BID
N = 33
Tofacitinib 15 mg BID
N = 49
Discontinued study
N = 13
Lack of efficacy 5
Adverse event 3
Lost to follow-up 1
Protocol violation 2
Consent withdrawn 2
Discontinued study
N = 11
Lack of efficacy 6
Adverse event 2
Lost to follow-up 0
Protocol violation 1
Consent withdrawn 2
Discontinued study
N = 7
Lack of efficacy 5
Adverse event 0
Lost to follow-up 0
Protocol violation 0
Consent withdrawn 2
Discontinued study
N = 2
Lack of efficacy 2
Adverse event 0
Lost to follow-up 0
Protocol violation 0
Consent withdrawn 0
Discontinued study
N = 4
Lack of efficacy 1
Adverse event 2
Lost to follow-up 0
Protocol violation 1
Consent withdrawn 0
Completed Week 8
N = 35 (72.9%)
Completed Week 8
N = 20 (64.5%)
Completed Week 8
N = 26 (78.8%)
Completed Week 8
N = 31 (93.9%)
Completed Week 8
N = 45 (91.8%)
