# Supplementary figures and images for: Randomized trial of tofacitinib in active ulcerative colitis: analysis of efficacy based on patient-reported outcomes
Source: BMC Gastroenterol. 2015 Feb 5;15:14. doi: 10.1186/s12876-015-0239-9 (PMC4323227; doi:10.1186/s12876-015-0239-9)

a

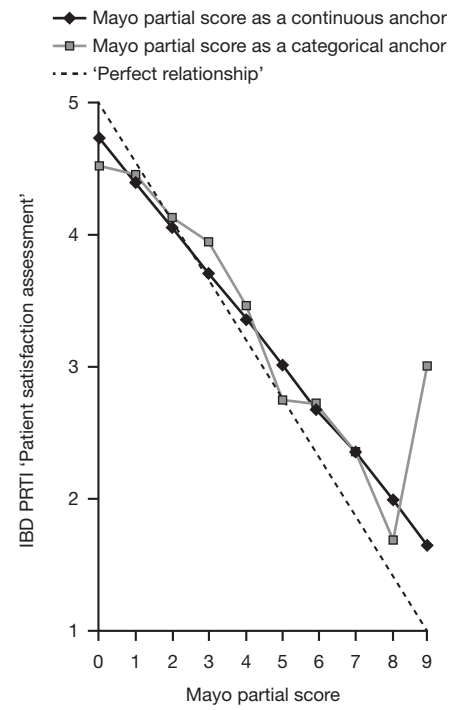

b

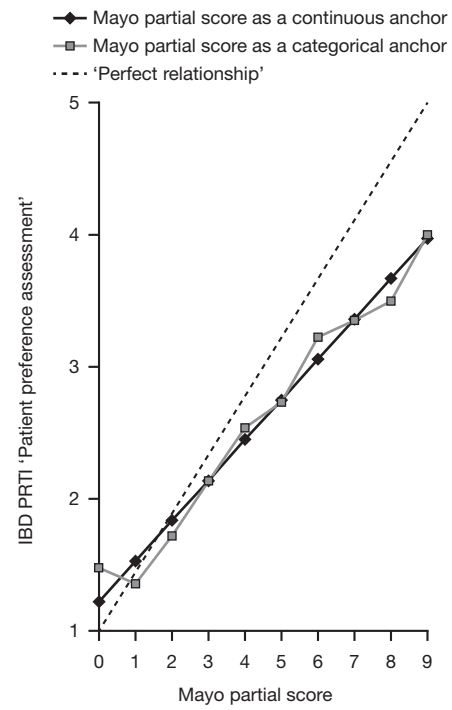

c

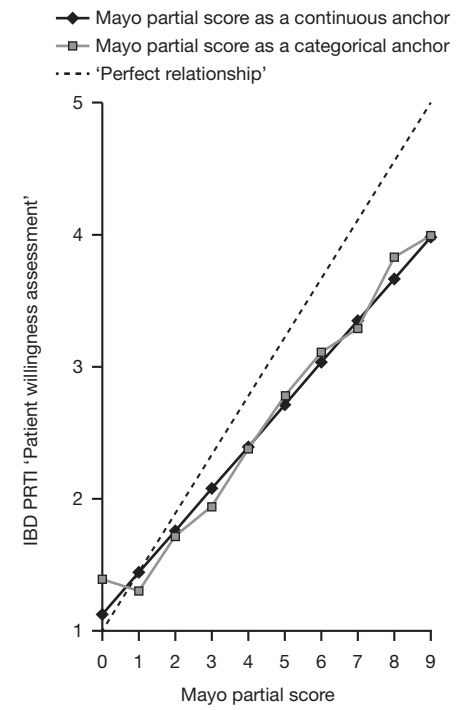

Supplement: Additional file 5: — Relationship between Patient-Reported Treatment Impact and clinical disease activity in ulcerative colitis. Relationship between the components of the IBD Patient-Reported Treatment Impact (all patients) versus the Mayo partial score. IBD PRTI item scores: Patient satisfaction assessment; Extremely dissatisfied with study drug = 1, Dissatisfied with study drug = 2, Neither satisfied nor dissatisfied with study drug = 3, Satisfied with study drug = 4, Extremely satisfied with study drug = 5. Patient preference assessment; Definitely prefer study drug over prior treatment = 1, Slightly prefer study drug = 2, No preference = 3, Slightly prefer prior treatment = 4, Definitely prefer prior treatment = 5. Patient willingness assessment; Would definitely use study drug again = 1, Might use study drug again = 2, Not sure = 3, Might not use study drug again = 4, Would definitely not use study drug again = 5. [file 12876_2015_239_MOESM5_ESM.pdf]
